# High Resolution Melting Analysis Is a More Sensitive and Effective Alternative to Gel-Based Platforms in Analysis of SSR – An Example in Citrus

**DOI:** 10.1371/journal.pone.0044202

**Published:** 2012-08-30

**Authors:** Gaetano Distefano, Marco Caruso, Stefano La Malfa, Alessandra Gentile, Shu-Biao Wu

**Affiliations:** 1 Dipartimento di Scienze delle Produzioni Agrarie e Alimentari, University of Catania, Catania, Italy; 2 School of Environmental and Rural Science, University of New England, Armidale, Australia; United States Department of Agriculture, United States of America

## Abstract

High resolution melting curve analysis (HRM) has been used as an efficient, accurate and cost-effective tool to detect single nucleotide polymorphisms (SNPs) or insertions or deletions (INDELs). However, its efficiency, accuracy and applicability to discriminate microsatellite polymorphism have not been extensively assessed. The traditional protocols used for SSR genotyping include PCR amplification of the DNA fragment and the separation of the fragments on electrophoresis-based platform. However, post-PCR handling processes are laborious and costly. Furthermore, SNPs present in the sequences flanking repeat motif cannot be detected by polyacrylamide-gel-electrophoresis based methods. In the present study, we compared the discriminating power of HRM with the traditional electrophoresis-based methods and provided a panel of primers for HRM genotyping in Citrus. The results showed that sixteen SSR markers produced distinct polymorphic melting curves among the *Citrus spp* investigated through HRM analysis. Among those, 10 showed more genotypes by HRM analysis than capillary electrophoresis owing to the presence of SNPs in the amplicons. For the SSR markers without SNPs present in the flanking region, HRM also gave distinct melting curves which detected same genotypes as were shown in capillary electrophoresis (CE) analysis. Moreover, HRM analysis allowed the discrimination of most of the 15 citrus genotypes and the resulting genetic distance analysis clustered them into three main branches. In conclusion, it has been approved that HRM is not only an efficient and cost-effective alternative of electrophoresis-based method for SSR markers, but also a method to uncover more polymorphisms contributed by SNPs present in SSRs. It was therefore suggested that the panel of SSR markers could be used in a variety of applications in the citrus biodiversity and breeding programs using HRM analysis. Furthermore, we speculate that the HRM analysis can be employed to analyse SSR markers in a wide range of applications in all other species.

## Introduction

The citrus species are widespread crops in over 100 countries and their production has experienced continuous growth in the last decade with a total annual production over 120 million tons [1]. Their nutritional, medicinal, and refreshing fragrance values have been appreciated since ancient times, and the study of their evolution is a complex process because of the great diversity and the apparently distant centers of origin [2]. *Citrus* taxonomy is very complex mainly due to sexual compatibility between *Citrus* and related genera, the high incidence of nucellar polyembryonic reproduction, the high frequency of bud mutations, the long history of cultivation, and worldwide distribution [3]. The level of genetic variability in *Citrus* has been evaluated by a number of studies using various molecular markers [3]–[12]. Among those, SSR markers were widely used for genetic diversity assessment, phylogenetic studies, genome mapping and population structure analysis [9], [11]–[13].

SSRs have been highly popular genetic markers for last two decades because of their hypervariability, codominance, multiallelic nature, high reproducibility, extensive genome coverage and the amenability to automation and high throughput genotyping [14], [15]. They have been used extensively in plant genetics, biodiversity and cultivar identification, and are constantly isolated and characterized in a wide range of economically important plant species. The traditional protocols used for SSR genotyping employ loci-specific primers to PCR amplify the DNA fragment containing nucleotide repeats, and the PCR products are separated using laborious polyacrylamide gels involving radioactivity detection, use of carcinogenic DNA stains or tedious silver staining, or automated capillary electrophoresis (CE) system with fluoro-labelled primers. Although CE significantly improved the throughput and automatisation [14], the procedure requires post-PCR handling and is costly. In addition, the nucleotide variations such as single nucleotide polymorphisms (SNPs) in the flanking sequences of repeat motif cannot be detected as the electrophoresis based methods only differentiate the genotypes with length polymorphisms. Therefore, more potentials of the marker system have been restrained by the approaches to analyze the markers.

Recently, high resolution melting analysis (HRM) has been identified as a powerful method that can be applied to analyze the genetic variations including SNPs, insertions or deletions (INDELs), and methylations of DNA in PCR amplicons [16]–[17]. It is a measurement of fluorescence change accompanied by the double strand DNA melting using a saturated DNA intercalating dye and a highly precise optical detection system, now usually attached to the realtime PCR machines. A subtle variation in the DNA sequence leads to detectable change of melting curve, and thus the allelic differences among PCR amplicons are distinguished. HRM analysis was proved to be a relatively efficient, accurate and inexpensive method to detect the polymorphisms especially SNPs [18], [19]. The approach has already been used to study genetic variability of plant species, for instance, apple, barley, grapevine, olive, almond, pepper and sweet cherry [19]–[25]. Lately, this approach has also been used in genetic mapping and biodiversity analysis [21], [26], [27]. HRM approach has also been applied to analyze microsatellite markers in a few species [22], [23], [25], [28], [29]. However, only a handful of genotypes as well as markers were tested and no conclusion can be drawn whether HRM can be an alternative to electrophoresis-based methods for microsatellite detection. Furthermore, no report has shown that HRM can distinguish other nucleotide variations in the flanking sequence of the nucleotide repeats although this has been predicted [29].This detection is apparently impossible by other typically used analysis methods for microsatellite amplicons such as the capillary or conventional polyacrylamide gel electrophoresis-based methods which only separate fragments in different sizes.

We hypothesize that HRM can efficiently and accurately distinguish the variation of nucleotide repeats in microsatellite markers, and the concurrent nucleotide variations mainly SNPs in the sequence flanking repeat motif can also be identified. The aim of this study was to evaluate whether HRM can be an alternative to electrophoresis-based methods such as capillary electrophoresis, and whether it can distinguish more polymorphisms than just repeat variations in citrus species. The outcomes of the work will also provide a reliable and robust set of SSRs to assess genetic diversity and conduct fingerprinting through HRM platform.

## Results

Twenty four primer pairs amplifying fragments less than 250 bp were chosen from the study of Luro [11]. Among those, 16 primer pairs produced clear genotyping profiles generated from both HRM and capillary analysis (Table S1), which were used in this study. While the other 8 primer pairs produced distinct HRM curves, the capillary electrophoresis profiles were obscure thus difficult to interpret. Therefore, these markers were excluded in this study.

All the 16 SSR markers produced polymorphic melting curves among the *Citrus spp* investigated through HRM analysis. In total, 66 alleles were detected altogether (Table 1) with the average of 4.1 alleles per marker, which identified the average of 5 genotypes in the population under investigation. In addition, the mean of the polymorphism information content (PIC) of these 16 markers was 0.493, the average major allele frequency was 0.581, and observed heterozygosity was 0.463. Among those, marker 482, representing a highest polymorphic marker, had 8 alleles which identified 10 genotypes and its PIC was 0.735, while 338, being a least polymorphic marker, had only 2 alleles which identified 2 genotypes in the analyzed population and its PIC was 0.374.

**Table 1:** Comparison of the allele and genotype information of the markers between CE and HRM analyses.

Marker	Major Allele Frequency		Genotype No		Allele No		Observed Heterozygosity		PIC	
	HRM	CE	HRM	CE	HRM	CE	HRM	CE	HRM	CE
16	0.333	0.567	8	5	4	3	0.800	0.533	0.692	0.519
21	0.433	0.600	7	5	7	4	0.467	0.333	0.730	0.475
34	0.500	0.900	4	3	4	3	0.133	0.133	0.500	0.175
92	0.833	0.900	4	3	4	2	0.267	0.067	0.282	0.164
93	0.667	1.000	4	1	3	1	0.133	0.000	0.445	0.000
115	0.533	0.533	5	5	4	4	0.733	0.733	0.521	0.521
116	0.433	0.433	7	7	4	4	0.667	0.667	0.638	0.638
137	0.933	0.967	3	2	3	2	0.133	0.067	0.123	0.062
203	0.700	0.900	4	2	4	2	0.267	0.200	0.450	0.164
338	0.533	0.533	2	2	2	2	0.933	0.933	0.374	0.374
430	0.667	0.667	4	4	3	3	0.133	0.133	0.383	0.383
482	0.433	0.433	10	10	8	8	0.733	0.733	0.735	0.735
818	0.600	0.900	4	3	3	2	0.067	0.067	0.466	0.164
1210	0.400	0.867	5	4	7	3	0.867	0.133	0.643	0.221
1388	0.567	0.900	5	3	3	2	0.600	0.067	0.478	0.164
1527	0.667	0.667	4	4	3	3	0.467	0.467	0.433	0.433
**Mean**	**0.581**	**0.735**	**5**	**3.9**	**4.1**	**3**	**0.463**	**0.329**	**0.493**	**0.325**

The same citrus genotypes were analyzed by capillary electrophoresis. In total, 48 alleles were detected (Table 1) with the average of 3 alleles per marker which identified the average of 3.9 genotypes in the population investigated. The mean of the PIC of these 16 markers analyzed by capillary electrophoresis was 0.325, the average major allele frequency was 0.735, and observed heterozygosity was 0.329. Same as the HRM analysis, marker 482, representing a highest polymorphic marker, had 8 alleles which identified 10 genotypes and its PIC was 0.735. 338 was also a least polymorphic marker following the capillary electrophoresis analysis.

**Figure 1:**
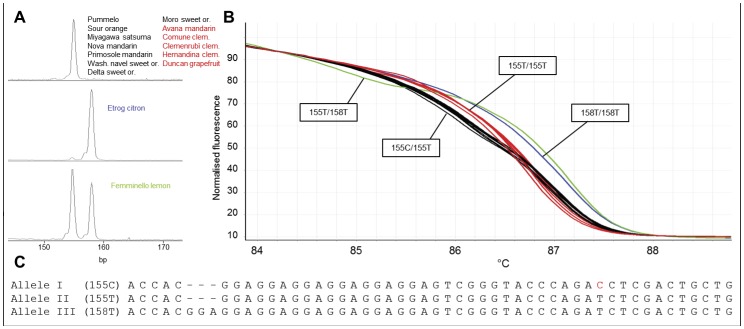
CE and HRM profiles of 15 citrus genotypes analyzed with the marker 1388. The HRM analysis revealed a novel genotype, 155C/155T, which was not recognized by CE. **A**. CE profiles of 3 distinct genotypes with 2 alleles (155 bp and 158 bp). Fragment size includes the M13 primer tail (19 bp). **B**. HRM curves showing 4 genotypes in normalized melting plot, two homozygous with single and two heterozygous with double melting phases. A new allele was detected in the analysis. Curves with different color represent different genotypes which are also shown in **A**. The exact genotypes are indicated in the rectangular boxes. **C**. Alignment of the 3 identified alleles by the bidirectional sequencing which identified a new allele which includes a SNP (red).

**Figure 2:**
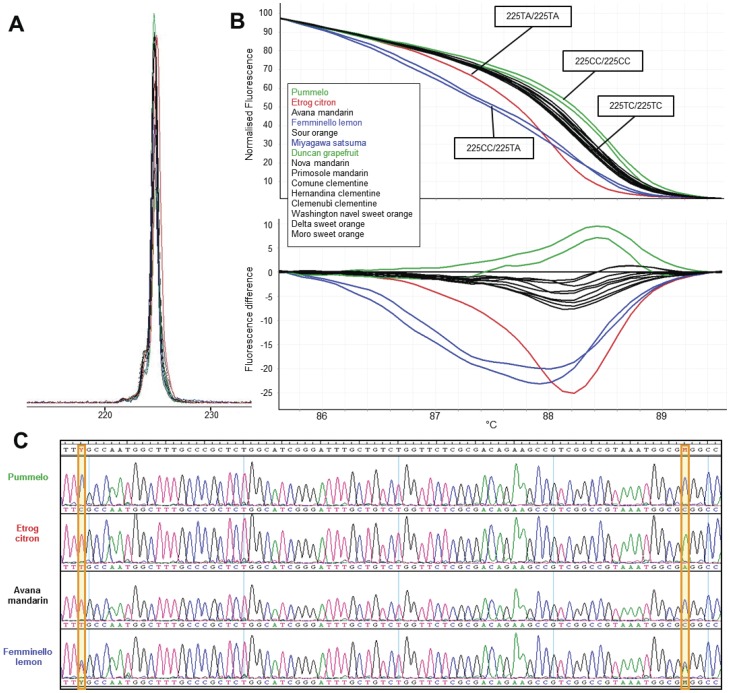
CE and HRM profiles, and sequence alignment of genotypes/alleles analyzed with the marker 93. A monomorphic marker in the population under study is shown polymorphic by HRM analysis. **A**. Monomorphic CE profile obtained from the analysis of 15 citrus genotypes. **B**. Polymorphic HRM melting curves showing 4 genotypes in normalized melting plot (upper) and difference melting curve (lower). The curves with different color represent different genotypes which are also shown in **A**. The exact genotypes are indicated in the rectangular boxes. **C**. Sequence alignment of the amplicons from 4 distinct genotypes. Two SNPs are shown and highlighted in the yellow boxes.

Out of the 16 primers, 10 primers showed more genotypes by HRM analysis than capillary electrophoresis, which were due to the presence of SNPs in the sequences flanking the SSR repeats (Table S1). These SNPs were confirmed by sequencing the amplicons of these markers. For example, marker 1388 showed only 2 alleles and 3 genotypes discriminated by CE analysis. However, when the marker was subjected to HRM analysis, 3 alleles and consequently 4 genotypes were shown as shown in Figure 1. Following sequencing of the amplicons, it was recognized that a C/T SNP in the sequences flanking nucleotide repeats contributed the increase of allele number and the polymorphism of the analyzed population. Interestingly, the marker 93 was considered monomorphic with CE analysis with a single peak at the size of 225 bp. However, 4 distinct melting curves were obtained by HRM analysis as the result of two SNPs (C/T and A/C) in the repeat flanking regions which were shown by sequencing of the amplicons of the individual genotypes (Figure 2).

**Figure 3:**
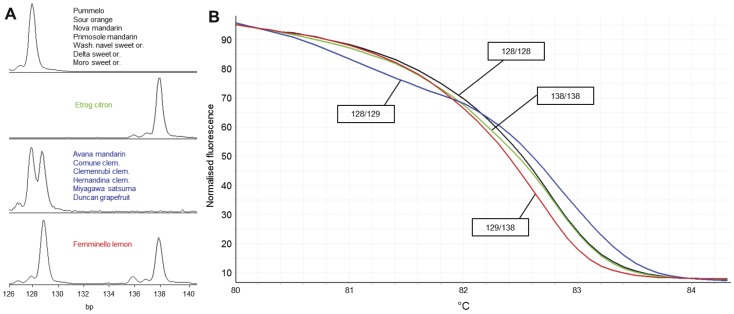
CE and HRM profiles of the genotypes analyzed with the marker 1527. The HRM analysis produced consistent result with CE when only length polymorphisms were present in the amplicon. **A**. CE profiles of 4 different genotypes, two homozygous (128/128 bp, and 138/138 bp) and two heterozygous (128/129 bp, and 129/138 bp). Fragment size includes the M13 primer tail (19 bp). **B**. HRM melting profiles showing 4 distinct genotypes in normalized melting plot which are consistent with the CE results. The exact genotypes are indicated in the rectangular boxes.

**Figure 4:**
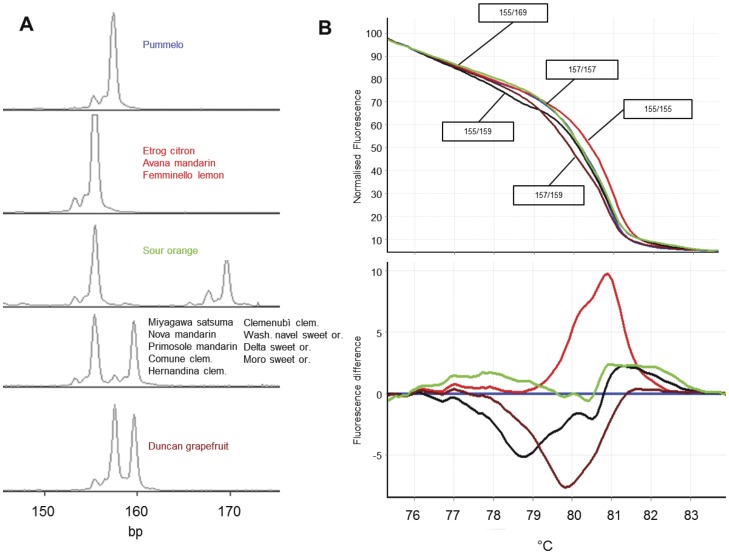
CE and HRM profiles of the genotypes analyzed with the marker 115. While normalized HRM plot shows ambiguous genotype separation, further analysis with difference curve resolves the genotypes clearly. **A**. CE profile of 5 different genotypes. Four different alleles (155 bp, 157 bp, 159 bp and 169 bp) form 2 homozygous and 3 heterozygous genotypes. Fragment size includes the M13 primer tail (19 bp). **B**. Normalized HRM melting curves showing 4 genotypes - two ambiguous genotypes not resolved. **C**. Difference plot showing 5 distinct genotypes which resolved two ambiguous genotypes shown in B, which was consistent with CE results. The exact genotypes are indicated in the rectangular boxes.

**Figure 5:**
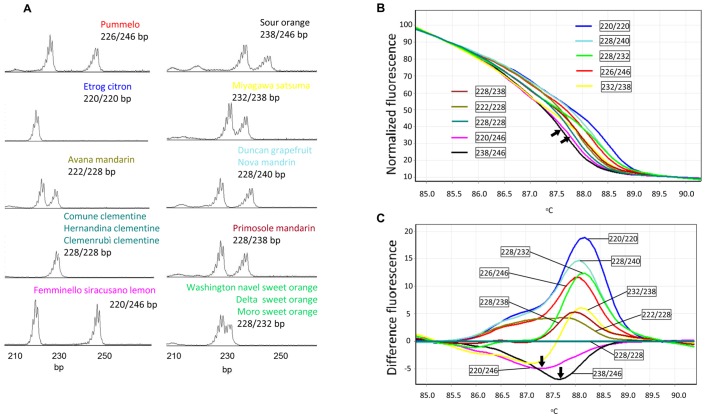
CE and HRM profiles of the genotypes analyzed with the marker 482 showing 10 genotypes shown by both CE and HRM analysis. A . CE profiles of 10 different genotypes. Seven different alleles (220 bp, 222 bp, 228 bp 232 bp, 238 bp, 240 bp and 246 bp) form 2 homozygous and 8 heterozygous genotypes. Fragment size includes the M13 primer tail (19 bp). **B**. Normalized HRM plot showing two similar melting curves produced from different genotypes (arrows). **C** difference plot - two ambiguous genotypes were resolved (arrows) thus 10 genotypes were distinguished which was in agreement with the CE results. The exact genotypes are indicated in the rectangular boxes.

As for the SSR markers without SNPs present in the flanking region, HRM also gave distinct melting curves which discriminated same genotypes shown in CE analysis. As shown in Table 1, all the rest of 6 markers showed the same numbers of alleles and genotypes with both HRM and CE analyses. An example of this was the marker 1527 in which four genotypes were detected by both HRM and CE analyses (Figure 3). It was noted that in some cases, the HRM produced similar normalized melting curves for two distinct SSR genotypes. However, when difference plot was used to view the melting curves for these genotypes, they were distinctly distinguished, for example in the marker 115 (Figure 4). Similarly, marker 482 produced 10 melting curves representing 10 genotypes of 15 citrus individuals. Due to higher number of polymorphisms, the normalized plot did not differentiate some of the genotypes. Difference plot, however, distinguished all the 10 genotypes from each other (Figure 5).

**Figure 6:**
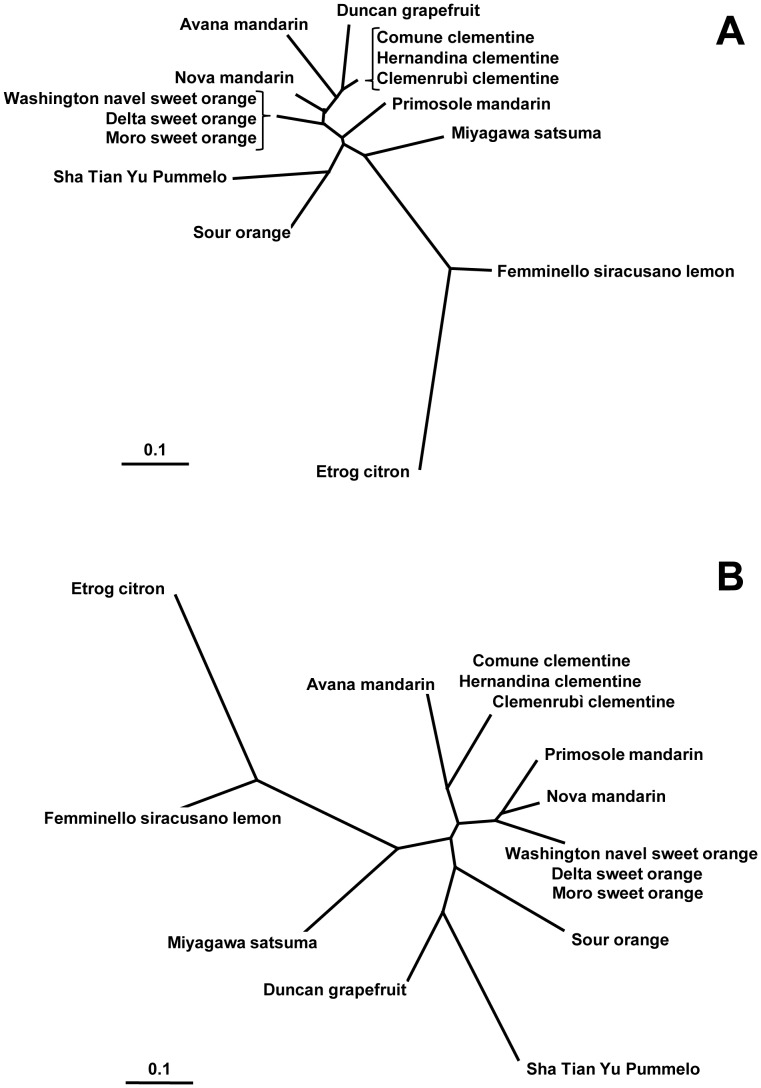
Dendrogram representing the structure of genetic diversity and relationships among 15 citrus genotypes by CE (A) and HRM (B). Genetic distance analysis allowed discrimination of most of the 15 citrus genotypes except those belonging to the same species (3 sweet orange and 3 clementine varieties). Citrus genotypes sharing same parental origin clustered in the same group. This fact is especially evident in HRM dendrogram in which all mandarin and clementine genotypes (except Satsuma) are grouped together. The genetic distance was calculated by Powermarker (shared alleles), and clustering was produced using Neighbor-joining method.

HRM and CE analyses allowed discriminating most of the 15 citrus genotypes except for those belonging to the same species (3 sweet orange and 3 clementine varieties). In the dendrograms shown in Figure 6, genotypes sharing same parental origin were clustered in the same group, whereas the ones with different origin were clearly separated into different groups. In dendrogram constructed with HRM data, genetic distance analysis generated three main branches, i.e., the first group including ‘Avana’ mandarin, clementines on the one side and the hybrids from them ‘Nova’, ‘Primosole’ and sweet oranges on the other side, the second group including ‘Duncan’ grapefruit, ‘Sha Tian Yu’ pummelo and sour orange, and the third group including ‘Etrog’ citron, ‘Femminello siracusano’ lemon and ‘Miyagawa’ satsuma.

## Discussion

HRM has been proved an efficient, and cost-effective approach to detect sequence variations such as SNPs in humans, plants and microorganisms. In plants, this approach has been applied to detect SNPs and SSRs which were used for genotype identifications and genetic mapping[19]–[27]. Potentially, it can also be used for mutation scanning [30]. The time and costs of the analysis is similar to conventional PCR but it omits the need for post-PCR separation to visualize the genotypes by means of, for example, gel electrophoresis. Although a few reports have described the application of HRM in discrimination of SSR genotypes, the merits of using this method have not been fully reported [22], [23], [25], [28], [29]. Furthermore, comprehensive comparisons between the HRM and conventional electrophoresis-based analyses of SSRs markers are still lacking. In this study, we analyzed 16 SSR markers adopted from the study of Luro [11] using HRM and the results were compared with those produced from capillary electrophoresis analysis. The amplicons were sequenced to confirm the results from HRM analysis and to reveal *de novo* SNPs present in the fragments. The results showed that HRM is not only a method suitable for discriminating SSR genotypes, but also a more accurate approach which can detect more alleles that contain SNPs in the sequences flanking SSR repeat motifs.

HRM has been applied to analyze SSR markers in a few studies [22], [23], [25], [28], [29]. However, some limitations were recognized that related to the sequence complexity of microsatellite and high number of alleles in the analyzed population. HRM was thought to be effective for low complex SSRs with a low number of alleles to ensure reliable interpretations of the melting curve profiles of the genotypes [28]. Also, HRM analysis would lose the power if multilocus markers are present, or PCR amplification is not specific. In our study, HRM was demonstrated a good alternative to the electrophoresis-based method despite the limitations already recognized. Basically, it detected all the genotypes that were present in the capillary electrophoresis, and uncovered more polymorphisms due to the presence of SNPs in the region flanking the SSR repeats. Therefore, HRM analysis produced higher numbers of alleles/genotypes, and thus PIC values and observed heterozigosity in the population. The use of HRM may be helpful to overcome the limits of electrophoresis based SSR analyses in cases of homoplasy that can lead to insufficient information to interpret the genetic distances among the genotypes due to the presence of SNPs in the amplicons [31]. Homoplasy in microsatellite alleles has been observed [32] and is thought to increase at interspecific or intergeneric levels [33]. Furthermore, the occurrence of homoplasy in citrus SSR markers has been previously reported [31]. Sequencing of some microsatellite loci showed that the SSR motifs were generally conserved among species and genera, but many variations in flanking sequences were observed. Therefore, alleles of the same size are not always characterized by identical sequence content, as shown in the present study and previous report [34].

The HRM analysis allowed discrimination of the genotypes at the species level for the 15 citrus varieties/hybrids by using the panel of 16 SSR markers adopted from Luro [11]. However, genotypes were not distinguishable at intraspecific level using this panel of markers. These varieties/hybrids are derivatives of different citrus genetic sources. Many of the sweet orange, grapefruit, lemon, clementine mandarin, and satsuma mandarin accessions were originated through somatic mutations which altered some horticultural characters but genetic similarity is still very high [4], [7]. This has been considered the main reason why molecular marker systems used so far are not effective in discriminating genotypes of such origin at intraspecific level. Hence, thorough investigations of the genetic alterations in the varieties of citrus at intraspecific level are warranted to establish specific fingerprinting profiles for the each individual variety. As HRM has increased power to reveal more polymorphisms in the citrus population, the sequencing of the citrus genome in more depth with wider sources will assist eventual resolution of the molecular differences among the intraspecific genotypes. Therefore, we expect HRM analysis will provide more power to create the fingerprinting profile at intraspecific level.

The two dendrograms obtained by CE and HRM data gave consistent results. In both, genotypes with same parental origin were generally clustered in the same groups. However, some genotypes of the same parental origin clustered closer with HRM data than CE data (i.e. Duncan grapefruit and Pummelo; Avana mandarin and clementines). Dendrogram based on HRM data also showed a clearer separation of the genotypes than that based on CE data owing to the additional SNPs detected by the HRM analysis. The genetic distance analysis of HRM data clustered the 15 citrus genotypes under investigation into 3 major groups, each containing one of the ancestral true species (pummelo, citron and mandarin). The only exception was that satsuma mandarin was unexpectedly clustered in the branch close to lemon and citron. It was noted, however, that haplotypes formed by a SNP and SSR in 3 markers were unique to this cultivar but not present in other mandarin oranges. We are uncertain whether this result is true representation of its genetic distance from other mandarins, oranges and close varieties, or analysis bias may be generated from the selection of markers by chance. Another possibility is that the limited representation of citrus diversity in the present study can be an origin of unexpected genetic distance between Satsuma and the mandarin and orange group. Nevertheless, our clustering result is supported in general by many studies that verified the hypothesis that only 3 *Citrus* types, namely the citron, the mandarin and the pummelo, constituted true or valid species [3], [7], [9], [35]–[37].

The present study produced the HRM profiles for a panel of 16 SSR markers selected from the study of Luro [11] in a population including the genotypes covering wide range of citrus species. The results have shown the clear separation of the genotypes at the species level by these markers. This suggests that this panel of SSR markers can be applied to identify different species and hybrids using the HRM approach without the requirement of post PCR procedure as has been required in traditional microsatellite analysis. Therefore, the information generated here can be used in other biodiversity and breeding programs in the international citrus community. Moreover, the method presented in this study, i.e., HRM analysis of SSR markers adapted from publications or developed *de novo*, can be widely used in all the plant and animals species in the areas such as biodiversity analysis, genetic mapping and breeding programs.

In spite of many advantages of the HRM analysis on SSR markers, we should not overlook the limitation of HRM analysis. When the genotype number of an SSR marker becomes high, the melting curves produced by some distinct genotypes tend to be similar. While the sensitivity of the assay can be increased by using HRM difference plotting, the variations of the melting curves posed among the same genotypes increase such that the genotypes of the individuals can be inconclusive. This can be expected when the population under investigation is large and involves many species which lead to a large number of alleles. To address this limitation, the ambiguous genotypes can be resolved by mixing them equally with a sample of known homozygous genotypes and then performing HRM analysis [19], [25]. However, this undoubtedly increases the workload of the assay and post-PCR operation has to be carried out.

The study presented here has shown that HRM is not only an efficient and cost-effective alternative of traditional electrophoresis-based method for SSR markers, but also a method to uncover more polymorphisms caused by SNPs present in the regions flanking nucleotide repeats. Consequently, more polymorphism and/or polymorphic haplotypes can be discriminated using this approach. It is also suggested that the panel of SSR markers can be used in a variety of applications in the citrus biodiversity and breeding programs worldwide by using HRM analysis established in this study. Furthermore, we believe that the HRM analysis can be employed to analyse SSR markers in a wide range of applications in all other taxa including plant and animal species. However, some limitations of the HRM analysis cannot be overlooked despite its advantages.

## Methods

### Plant Materials

A group of 15 genotypes belonging to 11 citrus species and hybrids was used as a population for analysis (Table 2). The leaves of these genotypes were collected from the germplasm collection at the ‘Primosole’ experimental farm of Catania University (Catania, Italy). All the trees used for this study were healthy and subjected to standard cultivation practices.

**Table 2:** Citrus genotypes used for EST- SSRs analysis by capillary electrophoresis and high resolution melting.

Common name/Cultivar	Tanaka system	Category	Origin
Sour orange	*C. aurantium* L.	Rootstock	Italy
Comune clementine	*C. clementina* Hort. ex Tan.	Mandarin	Italy
Hernandina clementine	*C. clementina* Hort. ex Tan.	Mandarin	Spain
Clemenrubì clementine	*C. clementina* Hort. ex Tan.	Mandarin	Spain
Sha Tian Yu pummelo	*C. grandis* (L.) Osbeck	Pummelo	China
Femminello siracusano lemon	*C. limon* (L.) Burm. f.	Lemon	Italy
Etrog citron	*C. medica* L.	Citron	Israel
Duncan grapefruit	*C. paradisi* Macf.	Grapefruit	USA
Nova mandarin	[(*C. paradisi* Macf. x *C. reticulata*) x *C. clementina*]	Mandarin	USA
Primosole mandarin	*C. unshiu* Marcov. x *C. reticulata*	Mandarin	Italy
Avana mandarin	*C. deliciosa* Ten.	Mandarin	Italy
Delta sweet orange	*C. sinensis* (L.) Osbeck	Valencia orange	USA
Moro sweet orange	*C. sinensis* (L.) Osbeck	Blood orange	Italy
Washington navel sweet orange	*C. sinensis* (L.) Osbeck	Navel orange	USA
Miyagawa satsuma	*C. unshiu* Marc.	Satsuma	Japan

### DNA Extraction

Total DNA was extracted from young leaves according to the protocol of Doyle and Doyle [38] as modified by Deng [39]. Briefly, fresh young leaves were ground to a fine powder in liquid nitrogen and incubated with CTAB extraction buffer (2% CTAB, 100 mM Tris–HCl, pH 8.0, 20 mM EDTA, pH 8.0, 1.4 M NaCl, 0.1% 2-mercapthoethanol and PVP-40T) at 65°C for 30 minute. Non-nucleic-acid substances were removed with chloroform : isoamyl alcohol and the DNA was precipitated with cold isopropanol and washed in 75% ethanol. The purified DNA was dissolved in TE buffer (10 mmol/L Tris–HCl, 0.1 mmol/L EDTA, pH 8.0) and RNA was removed by incubating the sample with DNase-free RNase A. Additional proteins, including RNase, were precipitated with ammonium acetate, and the DNA was collected by precipitation with ethanol and dissolved in TE buffer. DNA samples with absorbance ratios above 1.7 [40] were used for the analysis in this experiment and stored at −20°C until needed.

### Primer Screening

Primers were selected from EST-SSRs developed on ‘Nules’ clementine (*C. clementina* Hort. ex Tan.) by Luro [11]. 39 primers were used to amplify DNA of clementine and sour orange in order to evaluate the size and the band pattern in agarose gel (1.5%). Only the monolocus markers with fragment size shorter than 250 bp were used for further analysis (Table 3).

**Table 3:** A panel of markers selected for CE and HRM analyses of 15 citrus genotypes.

SSRname	EST Accessionnumber	Repeatmotif	Allele sizerange (bp)	SNP variationin amplicon	SNP positionin the EST	AmpliconΔG (kcal/mol)
16	DY264179	(AG)^11^	132/136	C/T	206	1.5
21	DY264533	(TC)^8^	214/238	T/A-T/C-C/A-G/C	370–421–487–518	0.0
34	DY265633	(TA)^6^	167/171	C/T	288	0.2
92	DY272212	(ATC)^5^	241/244	C/T	348	0.5
93	DY272212	(CTT)^5^	206	C/T-A/C	668–749	0.9
115	DY274953	(TA)^6^	136/140			0.5
116	DY274953	(AGA)^7^	248/254			−0.4
137	DY280434	(CAA)^5^	163/166	A/G	143	−0.9
203	DY283710	(CTT)^5^	201/202	T/G–C/T	480–560	−3.0
338	DY299973	(CTT)^11^	192/195			0.6
430	DY275609	(AAT)^7^N^15^(AGC)^7^	118/124			0.5
482	DY296883	(GA)^10^	201/227			0.8
818	DY287851	(TCT)^6^	130/133	C/T	183	0.6
1210	DY275216	(ATC)^5^	176/179	A/T-A/T	277–292	−0.1
1388	DY289396	(GGA)^6^	136/139	C/T	396	0.5
1527	DY292105	(TC)^6^	103/119			1.7

### PCR Amplifications

For capillary electrophoresis, PCR reaction included the two specific primers (0.3 µM) plus a labeled M13F primer (CAC GAC GTT GTA AAA CGA C, 0.13 µM), approximately 30 ng of template DNA, 0.2 mM dNTPs, 1× PCR buffer II, 2 mM magnesium chloride, and 1 U of MyTaq DNA polymerase (Bioline, Meridian Life Science, Memphis, USA). PCR was performed at 95°C for 12 min; followed by 35cycles of 95°C for 30 sec, 53°C for 30 sec, and 72°C for 45 sec; and one final cycle of 72°C for 15 min, on GeneAmp 9700 and 2700 amplifiers (Applied Biosystems, Foster City, CA, USA). For HRM analysis, PCR amplifications were performed in a total volume of 10 µL on a Rotor-Gene 6500 realtime PCR Thermocycler (Corbett Research, Sydney, Australia) and PCR reaction preparation was automated by a CAS1200 liquid handling system (Corbett Research). The reaction mixture contained 20 ng of genomic DNA, 1x PCR buffer (Bioline, Sydney, Australia), 2.5 mM MgCl_2_, 0.2 mM dNTP, 300 nM forward and reverse primers, 1.5 µM Syto® 9 (Invitrogen, Sydney, Australia), and 0.5 U Biotaq DNA polymerase (Bioline). The amplification was achieved by a touchdown PCR protocol: first denaturation at 95°C for 2 min, then 50 cycles denaturation at 95°C for 5 s, annealing and extension for 10 s at 60°C for the first cycle and thereafter at 0.5°C decrease each for 10 cycles, and a final extension at 72°C for 2 min.

### High Resolution Melting Analysis

HRM analysis followed previous studies [19]. Briefly, prior to melting steps, PCR products were denatured at 95°C for 5 s, and then annealed at 50°C for 30 s to randomly form DNA duplexes. HRM was performed as follows: pre-melt at the first appropriate temperature for 90 s, and melt at a ramp of 10°C in an appropriate temperature range at 0.1°C increments every 2 s. The fluorescent data were acquired at the end of each annealing step during PCR cycles and each of the HRM steps with automatic gain optimization. For data quality control, PCR amplification was analyzed through the assessment of the CT value, end point fluorescence level, and the amplification efficiency. The data from low quality amplification were removed from HRM analysis. In particular, runs with CT value of over than 30 were considered not suitable for the analysis; outliers having end point fluorescence less than 50% of average fluorescence of the samples and the data from samples with amplification efficiency lower than 1.4 were omitted from analysis. High resolution melting curve analysis was performed using the HRM analysis module. The melting data were normalized by adjusting start and end fluorescence signals, respectively, of all samples to the same levels. The data were recorded and analyzed using the Rotor-Gene 6500 series software (Corbett Research). HRM curve for each individual was visually scored. Genotypes were identified by examining normalized, difference and derivative melt plots. To ensure good amplification of the fragments, the sequences of the SSR markers were submitted to the online secondary structure profiling software DINAMelt to determine the folding characteristics of the sequences and their suitability for HRM analysis [41].

### Capillary Electrophoresis

An aliquot of 0.5–2 µl of PCR product (depending on the performance of amplification of each primer pair) was mixed with 10 µl of formamide and 0.35 µl of LIZ-500 size standard and denatured at 95°C for 5 min. Up to three PCR products labelled with FAM, PET, or NED were pooled before separation in the ABI 310 Genetic Analyzer (Applied Biosystems) and analysis was conducted using Genemapper 4.0 software.

### DNA Sequencing and SNP Identification

The HRM-SSRs showing different results from CE-SSR analysis were sequenced using an ABI310 genetic analyzer (Applied Biosystems). Fragments amplified from genomic DNA were bidirectional sequenced to eliminate sequencing errors. PCR products were purified using a PCR purification kit following the protocol provided by the manufacturer (Bioline). Seqman software (DNAstar, Madison, WI, USA) was used for contig assembly and SNP identification. Sequences with a mixture of alleles of unequal lengths were reconstructed manually or using Champuru 1.0 software [42]. Haplotypes were estimated based on bidirectional sequencing, homology with sequences already deposited in the NCBI dbEST database, and on known ancestral relationships among the analyzed genotypes.

### Genetic Distance and Clustering

Genetic distances were calculated based on the proportion on shared alleles [43] using PowerMarker version 3.25 [44]. Clustering of the SSR data was undertaken by Neighbor Joining method, and viewed in TreeView [45]. PowerMarker was also utilized to determine the observed heterozygosity, the average of polymorphism information content (PIC value) [46] for each primer pair.

## Supporting Information

Table S1 **SSR and SNP haplotypes in 15 citrus genotypes as shown by CE and HRM analyses performed with 16 selected EST-SSRs markers.** Fragment sizes include the M13 tail (19 bp).
(PDF)

## References

[1] FAOSTAT (2012) Faostat statistical database. Food and Agriculture Organization of the United Nations. Available: http://faostat.fao.org/faostat via the Internet. Accessed 21 Apr 2012.

[2] Khan I (2007) Citrus genetics, breeding and biotechnology. Wallingford: CABI Publishing.

[3] Nicolosi E, Deng ZN, Gentile A, Malfa SL, Continella G, et al (2000) Citrus phylogeny and genetic origin of important species as investigated by molecular markers. Theor Appl Genet 100: 1155–1166.

[4] Luro F, Laigret F, Bove JM, Ollitrault P (1995) DNA amplified fingerprinting, a useful tool for determination of genetic origin and diversity analysis in *Citrus* . Hort Sci 30: 1063–1067.

[5] Herrero R, Asins MJ, Carbonell EA, Navarro L (1996) Genetic diversity in the orange subfamily Aurantioideae. I. Intraspecies and intragenus genetic variability. Theor Appl Genet 92: 599–609. 24166329 10.1007/BF00224564

[6] Fang DQ, Roose ML (1997) Identification of closely related citrus cultivars with inter-simple sequence repeat markers. Theor Appl Genet 95: 408–417.

[7] Federici CT, Fang DQ, Scora RW, Roose ML (1998) Phylogenetic relationships within the genus *Citrus* (Rutaceae) and related genera as revealed by RFLP and RAPD analysis. Theor Appl Genet 94: 812–822.

[8] Moore GA (2001) Oranges and lemons: Clues to the taxonomy of *Citrus* from molecular markers. Trends Genet 17: 536–540. 11525837 10.1016/s0168-9525(01)02442-8

[9] Barkley NA, Roose ML, Krueger RR, Federici CT (2006) Assessing genetic diversity and population structure in a citrus germplasm collection utilizing simple sequence repeat markers (SSRs). Theor Appl Genet 112: 1519–1531. 16699791 10.1007/s00122-006-0255-9

[10] Novelli VM, Cristofani M, Souza AA, Machado MA (2006) Development and characterization of polymorphic microsatellite markers for the sweet orange (*Citrus sinensis* L. Osbeck). Genet Mol Biol 29: 90–96.

[11] Luro FL, Costantino G, Terol J, Argout X, Allario T, et al (2008) Transferability of the EST-SSRs developed on Nules clementine (*Citrus clementina* Hort ex Tan) to other *Citrus* species and their effectiveness for genetic mapping. BMC Genomics 9: 287–299. 18558001 10.1186/1471-2164-9-287 PMC2435559

[12] Ollitrault P, Terol J, Garcia-Lor A, Berard A, Chauveau A, et al (2012) SNP mining in *C. clementina* BAC end sequences; transferability in the *Citrus* genus (Rutaceae), phylogenetic inferences and perspectives for genetic mapping. BMC Genomics 13: 13. 22233093 10.1186/1471-2164-13-13 PMC3320530

[13] Ollitrault F, Terol J, Pina JA, Navarro L, Talon M, et al (2010) Development of SSR markers from *Citrus clementina* (Rutaceae) BAC end sequences and interspecific transferability in *Citrus* . Am J Bot 97: 124–129. 10.3732/ajb.1000280 21616814

[14] Agarwal M, Shrivastava N, Padh H (2008) Advances in molecular marker techniques and their applications in plant sciences. Plant Cell Rep 27: 617–631. 18246355 10.1007/s00299-008-0507-z

[15] Kalia RK, Rai MK, Kalia S, Singh R, Dhawan AK (2011) Microsatellite markers: an overview of the recent progress in plants. Euphytica 177: 309–334.

[16] Herrmann MG, Durtschi JD, Wittwer CT, Voelkerding KV (2007) Expanded instrument comparison of amplicon DNA melting analysis for mutation scanning and genotyping. Clin Chem 53: 1544–1548. 17556647 10.1373/clinchem.2007.088120

[17] White HE, Hall VJ, Cross NCP (2007) Methylation-sensitive high resolution melting-curve analysis of the SNRPN gene as a diagnostic screen for Prader-Willi and Angelman syndromes. Clin Chem 53: 1960–1962. 17890436 10.1373/clinchem.2007.093351

[18] Liew M, Pryor R, Palais R, Meadows C, Erali M, et al (2004) Genotyping of single-nucleotide polymorphisms by high-resolution melting of small amplicons. Clin Chem 50: 1156–1164. 15229148 10.1373/clinchem.2004.032136

[19] Wu S-B, Wirthensohn MG, Hunt P, Gibson JP, Sedgley M (2008) High resolution melting analysis of almond SNPs derived from ESTs. Theor Appl Genet 118: 1–14. 18781291 10.1007/s00122-008-0870-8

[20] Chagne D, Gasic K, Crowhurst RN, Han Y, Bassett HC, et al (2008) Development of a set of SNP markers present in expressed genes of the apple. Genomics 92: 353–358. 18721872 10.1016/j.ygeno.2008.07.008

[21] Lehmensiek A, Sutherland M, McNamara R (2008) The use of high resolution melting (HRM) to map single nucleotide polymorphism markers linked to a covered smut resistance gene in barley. Theor Appl Genet 117: 721–728. 18553067 10.1007/s00122-008-0813-4

[22] Mackay JF, Wright CD, Bonfiglioli RG (2008) A new approach to varietal identification in plants by microsatellite high resolution melting analysis: application to the verification of grapevine and olive cultivars. Plant Methods 4: 8. 18489740 10.1186/1746-4811-4-8 PMC2396621

[23] Muleo R, Colao MC, Miano D, Cirilli M, Intrieri MC, et al (2009) Mutation scanning and genotyping by high-resolution DNA melting analysis in olive germplasm. Genome 52: 252–260. 19234553 10.1139/G09-002

[24] Jeong HJ, Jo YD, Kang BC (2010) Identification of *Capsicum* species using SNP markers based on high resolution melting analysis. Genome 53: 1029–1040. 21164536 10.1139/G10-094

[25] Ganopoulos I, Argiriou A, Tsaftaris A (2011) Microsatellite high resolution melting (SSR-HRM) analysis for authenticity testing of protected designation of origin (PDO) sweet cherry products. Food Control 22: 532–541.

[26] Wu S-B, Tavassolian I, Rabiei G, Hunt P, Wirthensohn M, et al (2009) Mapping SNP-anchored genes using high-resolution melting analysis in almond. Mol Genet Genomics 282: 273–281. 19526371 10.1007/s00438-009-0464-4

[27] Oliver RE, Lazo GR, Lutz JD, Rubenfield MJ, Tinker NA, et al (2011) Model SNP development for complex genomes based on hexaploid oat using high-throughput 454 sequencing technology. BMC Genomics 12: 77. 21272354 10.1186/1471-2164-12-77 PMC3041746

[28] Mader E, Lukas B, Novak J (2008) A strategy to setup codominant microsatellite analysis for high-resolution-melting-curve-analysis (HRM). BMC Genet 9: 69. 18980665 10.1186/1471-2156-9-69 PMC2588637

[29] Arthofer W, Steiner FM, Schlick-Steiner BC (2011) Rapid and cost-effective screening of newly identified micro satellite loci by high resolution melting analysis. Mol Genet Genomics 286: 225–235. 21847526 10.1007/s00438-011-0641-0

[30] Chen Y, Wilde HD (2011) Mutation scanning of peach floral genes. BMC Plant Biology 11: 96. 21605436 10.1186/1471-2229-11-96 PMC3120741

[31] Barkley N, Krueger R, Federici C, Roose M (2009) What phylogeny and gene genealogy analyses reveal about homoplasy in citrus microsatellite alleles. Plant Syst Evol 282: 71–86.

[32] Bhargava A, Fuentes FF (2010) Mutational dynamics of microsatellites. Mol Biotechnol 44: 250–266. 20012711 10.1007/s12033-009-9230-4

[33] van Oppen MJH, Rico C, Turner GF, Hewitt GM (2000) Extensive homoplasy, nonstepwise mutations and shared ancestral polymorphism at a complex microsatellite locus in Lake Malawi cichlids. Mol Biol Evol 17: 489–498. 10742041 10.1093/oxfordjournals.molbev.a026329

[34] Wu S-B, Franks T, Hunt P, Wirthensohn M, Gibson J, et al (2010) Discrimination of SNP genotypes associated with complex haplotypes by high resolution melting analysis in almond: implications for improved marker efficiencies. Mol Breed 25: 351–357.

[35] Scora RW (1975) On the history and origin of *Citrus* . Bull Torr Bot Club 102: 369–375.

[36] Scora RW (1988) Biochemistry, taxonomy and evolution of modern cultivated citrus. In: Goren R, Mendel K, editors. Citriculture. Tel Aviv: Proceedings of the International Society of Citriculture. 277–289.

[37] Barrett HC, Rhodes AM (1976) A numerical taxonomic study of affinity relationships in cultivated *Citrus* and its close relatives. Syst Bot 1: 105–136.

[38] Doyle JJ, Doyle JL (1987) A rapid DNA isolation procedure for small quantities of fresh leaf tissue. Phytochem Bull 19: 11–15.

[39] Deng ZN, Gentile A, Nicolosi E, Domina F, Vardi A, et al (1995) Identification of *in vivo* and *in vitro* lemon mutants by RAPD markers. J Hortic Sci 70: 117–125.

[40] Sambrook J, Russell DW (2001) Molecular cloning: a laboratory manual. Cold Spring Harbor: Cold Spring Harbor Laboratory.

[41] Markham NR, Zuker M (2005) DINAMelt web server for nucleic acid melting prediction. Nucleic Acids Res 33: W577–W581. 15980540 10.1093/nar/gki591 PMC1160267

[42] Flot JF (2007) Champuru 1.0: a computer software for unraveling mixtures of two DNA sequences of unequal lengths. Mol Ecol Notes 7: 974–977.

[43] Bowcock AM, Ruiz-Linares A, Tomfohrde J, Minch E, Kidd JR, et al (1994) High resolution of human evolutionary trees with polymorphic microsatellites. Nature 368: 455–457. 7510853 10.1038/368455a0

[44] Liu K, Muse SV (2005) Powermarker: An integrated analysis environment for genetic marker analysis. Bioinformatics 21: 2128–2129. 15705655 10.1093/bioinformatics/bti282

[45] Page RDM (1996) Treeview: An application to display phylogenetic trees on personal computers. Comput Appl Biosci 12: 357–358. 8902363 10.1093/bioinformatics/12.4.357

[46] Botstein D, White RL, Skolnick M, Davis RW (1980) Construction of a genetic linkage map in man using restriction fragment length polymorphisms. Am J Hum Genet 32: 314–331. 6247908 PMC1686077

