# Supplementary material for: High Resolution Melting Analysis Is a More Sensitive and Effective Alternative to Gel-Based Platforms in Analysis of SSR – An Example in Citrus
Source: PLoS One. 2012 Aug 30;7(8):e44202. doi: 10.1371/journal.pone.0044202 (PMC3431301; doi:10.1371/journal.pone.0044202)
Supplement: Table S1 — SSR and SNP haplotypes in 15 citrus genotypes as shown by CE and HRM analyses performed with 16 selected EST-SSRs markers. Fragment sizes include the M13 tail (19 bp). (PDF) [file pone.0044202.s001.pdf]

Table S1. Haplotype determination in 15 citrus genotypes as revealed by CE and HRM analyses

**CE analysis results**	**Marker**															
**Genotype**	**IN16**	**IN21**	**IN34**	**IN92**	**IN93**	**IN115**	**IN116**	**IN137**	**IN203**	**IN338**	**IN430**	**IN482**	**IN818**	**IN1210**	**IN1388**	**IN1527**
Pummelo	155/155	233/233	186/186	260/260	225/225	157/157	267/267	182/182	220/221	211/213	137/143	226/246	149/149	195/195	155/155	128/128
Etrog citron	155/155	255/255	190/196	262/262	225/225	155/155	273/273	182/182	220/220	211/211	140/140	220/220	152/152	198/198	158/158	138/138
Avana mandarin	151/153	257/257	186/186	260/260	225/225	155/155	267/276	182/185	220/220	211/213	143/143	222/228	149/149	195/195	155/155	128/129
Femminello lemon	153/155	257/257	186/190	260/262	225/225	155/155	273/276	182/182	220/220	211/213	140/140	220/246	149/152	195/198	155/158	129/138
Sour orange	153/155	233/257	186/186	260/260	225/225	155/169	267/276	182/182	220/221	211/213	140/140	238/246	149/149	195/195	155/155	128/128
Miyagawa satsuma	153/153	233/241	186/186	260/260	225/225	155/159	270/273	182/182	220/220	211/213	140/140	232/238	149/149	192/195	155/155	128/129
Duncan grapefruit	155/155	257/257	186/186	260/260	225/225	157/159	267/270	182/182	220/221	211/213	143/143	228/240	149/149	195/195	155/155	128/129
Nova mandarin	151/153	257/257	186/186	260/260	225/225	155/159	270/273	182/182	220/220	211/213	143/143	228/240	149/149	195/195	155/155	128/128
Primosole mandarin	151/153	233/233	186/186	260/260	225/225	155/159	270/273	182/182	220/220	211/213	140/143	228/238	149/149	195/195	155/155	128/128
Comune clementine	151/155	257/257	186/186	260/260	225/225	155/159	267/270	182/182	220/220	211/213	143/143	228/228	149/149	195/195	155/155	128/129
Hernandina clementine	151/155	257/257	186/186	260/260	225/225	155/159	267/270	182/182	220/220	211/213	143/143	228/228	149/149	195/195	155/155	128/129
Clemenrubì clementine	151/155	257/257	186/186	260/260	225/225	155/159	267/270	182/182	220/220	211/213	143/143	228/228	149/149	195/195	155/155	128/129
Washington navel sweet orange	155/155	233/257	186/186	260/260	225/225	155/159	270/270	182/182	220/220	211/213	143/143	228/232	149/149	195/195	155/155	128/128
Delta sweet orange	155/155	233/257	186/186	260/260	225/225	155/159	270/270	182/182	220/220	211/213	143/143	228/232	149/149	195/195	155/155	128/128
Moro sweet orange	155/155	233/257	186/186	260/260	225/225	155/159	270/270	182/182	220/220	211/213	143/143	228/232	149/149	195/195	155/155	128/128

**HRM analysis results**	**Marker**															
**Genotype**	**IN16**	**IN21**	**IN34**	**IN92**	**IN93**	**IN115**	**IN116**	**IN137**	**IN203**	**IN338**	**IN430**	**IN482**	**IN818**	**IN1210**	**IN1388**	**IN1527**
Pummelo	A/A	A/A	A/A	A/A	A/A	B/B	A/A	A/A	A/B	A/B	A/C	C/H	A/A	A/A	A/B	A/A
Etrog citron	B/B	C/C	C/D	C/D	B/B	A/A	C/C	A/A	C/C	A/A	B/B	A/A	C/C	D/D	C/C	C/C
Avana mandarin	C/D	D/D	A/A	A/A	C/C	A/A	A/D	A/C	D/D	A/B	C/C	B/D	A/A	B/C	B/B	A/B
Femminello lemon	B/D	D/F	A/C	A/D	A/B	A/A	C/D	A/A	C/D	A/B	B/B	A/H	B/C	E/F	B/C	B/C
Sour orange	A/D	B/E	A/A	A/B	C/C	A/D	A/D	A/A	A/B	A/B	B/B	F/H	A/A	B/C	A/B	A/A
Miyagawa satsuma	D/D	B/G	B/B	A/A	A/B	A/C	B/C	A/B	D/D	A/B	B/B	E/F	B/B	B/G	A/B	A/B
Duncan grapefruit	A/B	D/D	A/A	A/A	A/A	B/C	A/B	A/A	A/B	A/B	C/C	D/G	A/A	B/C	B/B	A/B
Nova mandarin	C/D	D/D	B/B	A/A	C/C	A/C	B/C	A/A	D/D	A/B	C/C	D/G	A/A	B/C	A/B	A/A
Primosole mandarin	C/D	A/B	B/B	A/B	C/C	A/C	B/C	A/A	D/D	A/B	B/C	D/F	A/A	B/C	A/B	A/A
Comune clementine	B/C	D/D	A/A	A/A	C/C	A/C	A/B	A/A	D/D	A/B	C/C	D/D	B/B	B/C	B/B	A/B
Hernandina clementine	B/C	D/D	A/A	A/A	C/C	A/C	A/B	A/A	D/D	A/B	C/C	D/D	B/B	B/C	B/B	A/B
Clemenrubì clementine	B/C	D/D	A/A	A/A	C/C	A/C	A/B	A/A	D/D	A/B	C/C	D/D	B/B	B/C	B/B	A/B
Washington navel sweet orange	A/B	B/E	B/B	A/A	C/C	A/C	B/B	A/A	D/D	A/B	C/C	D/E	A/A	B/C	A/B	A/A
Delta sweet orange	A/B	B/E	B/B	A/A	C/C	A/C	B/B	A/A	D/D	A/B	C/C	D/E	A/A	B/C	A/B	A/A
Moro sweet orange	A/B	B/E	B/B	A/A	C/C	A/C	B/B	A/A	D/D	A/B	C/C	D/E	A/A	B/C	A/B	A/A

**Allele identification**	Letters indicate alleles differing in size and in SNP															
	155C=A	233TTCG=A	186T=A	260C=A	225CC=A	155=A	267=A	182A=A	220TC=A	211=A	137=A	220=A	149T=A	195TA=A	155C=A	128=A
	155T=B	233TTCC=B	186C=B	260T=B	225TA=B	157=B	270=B	182G=B	221TC=B	213=B	140=B	222=B	149C=B	195AA=B	155T=B	129=B
	151T=C	255ATAG=C	190T=C	262C=C	225TC=C	159=C	273=C	185A=C	220TT=C		143=C	226=C	152C=C	195TT=C	158T=C	138=C
	153T=D	257ATAG=D	196T=D	262T=D		169=D	276=D		220GC=D			228=D		198AA=D		
		257TTCG=E										232=E		195AT=E		
		257ACCG=F										238=F		198AT=F		
		241TTCG=G										240=G		192TT=G		
												246=H		192AA=H		
